# Dietary Salt Restriction in Chronic Kidney Disease: A Meta-Analysis of Randomized Clinical Trials

**DOI:** 10.3390/nu10060732

**Published:** 2018-06-06

**Authors:** Carlo Garofalo, Silvio Borrelli, Michele Provenzano, Toni De Stefano, Carlo Vita, Paolo Chiodini, Roberto Minutolo, Luca De Nicola, Giuseppe Conte

**Affiliations:** 1 Division of Nephrology, University of Campania “Luigi Vanvitelli”, 80138 Naples, Italy; carlo.garofalo@hotmail.it (C.G.); dott.silvioborrelli@gmail.com (S.B.); michiprov@hotmail.it (M.P.); toni-ds@live.it (T.D.S.); carlovita90@gmail.com (C.V.); roberto.minutolo@unicampania.it (R.M.); luca.denicola@unicampania.it (L.D.N.); 2 Medical Statistics Unit, University of Campania “Luigi Vanvitelli”, 80138 Naples, Italy; paolo.chiodini@unicampania.it

**Keywords:** dietary salt restriction, blood pressure, proteinuria, chronic kidney disease

## Abstract

Background. A clear evidence on the benefits of reducing salt in people with chronic kidney disease (CKD) is still lacking. Salt restriction in CKD may allow better control of blood pressure (BP) as shown in a previous systematic review while the effect on proteinuria reduction remains poorly investigated. Methods. We performed a meta-analysis of randomized controlled trials (RCTs) evaluating the effects of low versus high salt intake in adult patients with non-dialysis CKD on change in BP, proteinuria and albuminuria. Results. Eleven RCTs were selected and included information about 738 CKD patients (Stage 1–4); urinary sodium excretion was 104 mEq/day (95%CI, 76–131) and 179 mEq/day (95%CI, 165–193) in low- and high-sodium intake subgroups, respectively, with a mean difference of −80 mEq/day (95%CI from −107 to −53; *p* <0.001). Overall, mean differences in clinic and ambulatory systolic BP were −4.9 mmHg (95%CI from −6.8 to −3.1, *p* <0.001) and −5.9 mmHg (95%CI from −9.5 to −2.3, *p* <0.001), respectively, while clinic and ambulatory diastolic BP were −2.3 mmHg (95%CI from −3.5 to −1.2, *p* <0.001) and −3.0 mmHg (95%CI from −4.3 to −1.7; *p* <0.001), respectively. Mean differences in proteinuria and albuminuria were −0.39 g/day (95%CI from −0.55 to −0.22, *p* <0.001) and −0.05 g/day (95%CI from −0.09 to −0.01, *p* = 0.013). Conclusion. Moderate salt restriction significantly reduces BP and proteinuria/albuminuria in patients with CKD (Stage 1–4).

## 1. Introduction

Non-dialysis chronic kidney disease (CKD) is recognized as a major global public health problem because of the widespread prevalence in the world, of about 10%, and the natural fate of progression to end-stage kidney disease (ESKD) in those that do not die before as consequence of the extraordinarily high cardiovascular (CV) and mortality risk [1,2,3].

However, the poor prognosis of CKD patients is modifiable. Hypertension and proteinuria are main surrogates of CV and renal outcomes that can be controlled by appropriate pharmacological and dietary interventions [4,5]. In particular, salt restriction has a great potential to attenuate these major complications of CKD. Indeed, extensive research in animal models and human studies suggest that dietary sodium restriction may slow the progression of renal disease and albuminuria [6,7,8,9]. Previous studies have shown that a low sodium diet can potentiate the effects of the renin–angiotensin–aldosterone system (RAAS) blockade and, therefore, decrease proteinuria and blood pressure (BP) as well [10,11].

Nevertheless, no randomized clinical trial (RCT) in CKD patients has evaluated the long-term effects of salt restriction on CKD progression, even though two post-hoc analyses have suggested beneficial effects on this endpoint [12,13]. A recent review by McMahon et al. was unable to select RCT assessing the long-term effects of sodium restriction on CV and all-cause mortality, in the whole spectrum of CKD, including patients undergoing renal replacement therapy, [14]. Therefore, this study could only evaluate the effect of sodium restriction on BP control, even though the reported significant reduction of BP levels was estimated by combining two different measurements obtained either in clinic or by 24-h monitoring. Finally, the data on proteinuria or albuminuria could not be meta-analyzed because of the few studies included.

Therefore, a clear evidence on the benefits of reducing salt in people with CKD, though of relevant clinical interest, is still lacking. The guidelines for dietary sodium intake in CKD are in fact based on expert opinion, observational studies, and extrapolation from general population studies [15]. Based on this background, we designed a systematic review and meta-analysis of RCTs assessing the efficacy of salt restriction on BP and albuminuria in CKD. This updated analysis can be helpful as it adds new insights for dietary recommendations in the high-risk population of CKD patients.

## 2. Materials and Methods

### 2.1. Search Strategies

The present review was conducted in accordance with the Preferred Reporting Items for Systematic reviews and Meta-Analyses (PRISMA) guidelines [16]. The searches for relevant articles published until 30 April 2018 was performed using PubMed and ISI Web of Science databases without language restriction. The following Medical Subject Headings (MeSH) and text words were used: “Chronic Renal Insufficiency”, “Kidney Diseases” “nephropathy”, “glomerular disease”, “chronic kidney disease”, “chronic kidney insufficiency”, “chronic kidney dysfunction”, “chronic renal dysfunction”, “Sodium Restricted Diet” “Sodium Chloride”, “sodium low”, “sodium high”, “sodium restriction”, “sodium reduction”, “sodium intake”, “sodium increase”, “salt restriction”, “salt reduction” (item S1). We screened references of articles and reviews found in research to identify other potentially relevant studies.

### 2.2. Study Selection

Criteria for inclusion were: (1) RCTs evaluating the effect of low versus high salt intake in people with non-dialysis or non-transplantation CKD; (2) Adults (≥18 years) with CKD (as defined by Kidney Disease: Improving Global Outcomes (KDIGO) Clinical Practice Guidelines [17]); (3) evaluating salt intake estimated by 24 h urinary sodium excretion (UNaV); (4) evaluating as outcome at least one of the following: change in BP (clinic or ambulatory monitoring); change in kidney function measures (albuminuria or proteinuria or GFR); change in body weight or fluid; change in UNaV and urinary potassium (UKV). We also evaluated adverse effects secondary to hypotension or worsening of renal function during low- and high-sodium diet.

Reduction in UNaV was calculated as the difference between UNaV at the end of each phase for cross-over studies, and the difference from baseline to the end of intervention for parallel studies. Other interventions, such as antihypertensive drugs, use of paricalcitol or dietary modifications, were considered when constant during low and high salt intake phases. In the case of overlapping studies in the same cohort, we examined those with the most complete information. Abstracts, letters to editors, commentaries, and reviews were excluded from our review.

The titles and abstracts, found with search strategy, were screened independently by two investigators (CG and SB). The full reports of potentially relevant studies were obtained and each paper was reviewed using predefined eligibility criteria. Any discrepancy between the two authors on study eligibility was resolved through discussion.

Data extraction was performed independently by two authors using standard data extraction forms.

### 2.3. Assessment of Risk of Bias in Included Studies

The risk of bias in the included RCTs was assessed by two reviewers by using the risk of bias assessment tool [18]. Nine items associated with the risk of bias were evaluated: adequate random sequence generation, allocation concealment, blinding of participants, blinding of assessment, incomplete outcome data adequately addressing, selective outcome reporting, other sources of bias, risk of carry over effect, and potential bias from confounding factors.

### 2.4. Statistical Analysis

We quantified the inter-rater agreement for study selection and quality assessment. To assess the effects of treatment the mean difference (MD) was used for final values of UNaV, clinic systolic blood pressure (SBP), clinic diastolic blood pressure (DBP), proteinuria and albuminuria in low and high salt subgroups while the unstandardized mean difference (UMD) was used to compare GFR, proteinuria, albuminuria, clinic SBP, clinic DBP, ambulatory SBP, ambulatory DBP, UNaV, UKV, body weight and total body water among two groups. When in the studies values were reported as median and interquartile range, we derived mean and standard deviation with method by Wan et al. [19]. Furthermore, when studies reported standard errors (SEs) instead of standard deviations (SDs), SDs were estimated based on the sample size (n), (SE = SD/√n). In cross-over studies, we determined the mean difference in outcomes as the difference between the end of low salt and high salt periods. Extracted results on estimate were pooled in the meta-analysis. We assumed a conservative approach in pooling results by using a random-effects model, which allows for variation of true effects across studies. We analyzed heterogeneity with the *I*^2^ statistic with 95% CI [20]. *I*^2^ values of 25%, 50%, and 75% correspond to cut-off points for low, moderate, and high degrees of heterogeneity. Sensitivity analyses were conducted to exclude that a study was exerting excessive influence on the heterogeneity [21]. Furthermore, univariate random-effects meta-regression and moderator analyses were performed to explore sources of heterogeneity where significant. Meta-regression was used to test difference between moderators. Restricted maximum likelihood estimators were used to estimate model parameters [22]. Begg’s rank correlation test and Egger’s linear regression were used to assess the publication bias [23]. Two-sided *p*-value < 0.05 is considered significant. 

Analyses were performed using PROMETA 2 (INTERNOVI, Cesena, Italy), STATA/SE 11 (Stata Corporation, College Station, TX, USA) and RStudio version 1.1442 (RStudio: Integrated development environment for R. Boston, US).

## 3. Results

### 3.1. Characteristics of Studies

**Figure 1:**
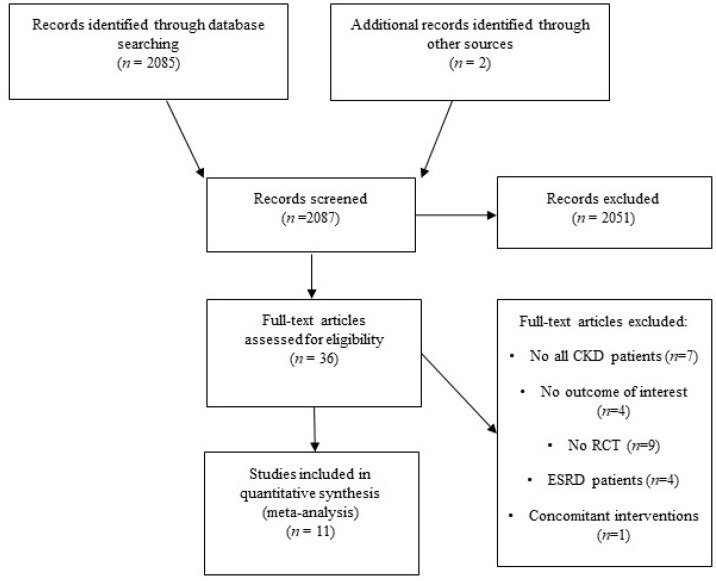
Flow-chart of study selection.

After screening titles and abstracts, 36 studies out of 2087 were considered. The full text of each article was reviewed by two authors, and 11 studies [11,23,24,25,26,27,28,29,30,31,32] were included in the present meta-analysis (Figure 1). Agreement of two reviewers was very good for study selection (Kappa = 0.934). Eleven main outcomes were evaluated: clinic SBP, clinic DBP, ambulatory SBP, ambulatory DBP, albuminuria, proteinuria, GFR, UNaV, UKV, body weight and total body water.

### 3.2. Patient Characteristics

Selected RCTs are summarized in Table 1. Overall, studies included information on 738 individuals (Stage 1–4). The sample size of these studies ranged from 14 to 302 participants. Three studies included Asian populations [24,26,28] while the others included Western populations. Difference in clinic and ambulatory blood pressure and renal function parameters are depicted in Table 2. The risk of bias was low in more recent studies and unclear in previous papers (Table 3).

### 3.3. Outcomes

#### 3.3.1. Urinary Sodium Excretion

All included trials evaluated sodium excretion in subjects treated with low- and high-sodium diet. Overall, UNaV was 104 mEq/day (95%CI, 76–131; *I*^2^: 98.0%) and 179 mEq/day (95%CI, 165–193; *I*^2^: 85.5%) in low- and high-sodium intake subgroups, respectively. Unstandardized overall mean difference in UNaV comparing low- and high-sodium diet was −80 mEq/day (95%CI, −107, −53; *p* < 0.001). Heterogeneity was high (*I*^2^: 94.65%, *p* < 0.001). In sensitivity analyses, any study had a considerable influence on overall value; furthermore, age, variation in body weight, diabetes, CKD stages (stages 3–4 vs. stages 1–2) and use of RAS inhibitors did not act as significant moderators. Only intervention duration (>4 weeks vs. ≤4 weeks) was moderator with a significant difference (*p* = 0.005). No publication bias was found (Begg’s test: *p* = 0.484; Egger’s test: *p* = 0.719).

#### 3.3.2. Systolic Blood Pressure

**Figure 2:**
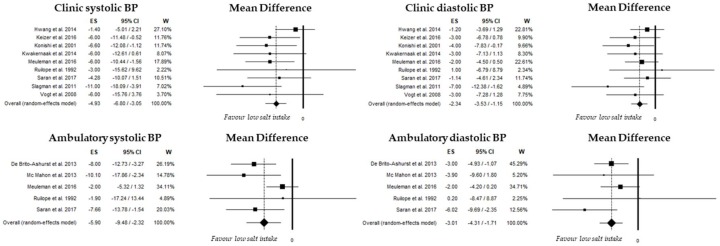
Mean difference of clinic and ambulatory blood pressure in low- and high-salt intake.

Nine studies evaluated difference in clinic systolic BP comparing low and high salt intake subgroups. Overall SBP was 129 mmHg (95%CI, 124–135; *I*^2^: 94.4%) and 135 mmHg (95%CI, 129–142; *I*^2^: 94.9%) in low- and high-sodium intake subgroups, respectively; differences in SBP were summarized in Figure 2 with an overall random-effect value of −4.9 mmHg (95%CI: −6.8, −3.1; *p* < 0.001). Heterogeneity was not significant (*I*^2^: 0%, *p* = 0.483). No publication bias was found (Begg’s test: *p* = 0.835; Egger’s test: *p* = 0.201).

A significant difference between low and high salt intake was also found in 5 RCTs evaluating ambulatory systolic BP (Figure 2) with an overall random-effect value of −5.9 mmHg (95%CI: −9.5, −2.3; *p* < 0.001). Heterogeneity was not significant among these studies (*I*^2^: 0.3, *p* = 0.405). No publication bias was found (Begg’s test: *p* = 1.0; Egger’s test: *p* = 0.424).

In the evaluation of moderator analyses, we tested the role of UNaV difference among low- and high-sodium intake groups on overall unstandardized mean of clinic and ambulatory SBP that was not significant.

#### 3.3.3. Diastolic Blood Pressure

Nine studies evaluated difference in clinic diastolic BP comparing low and high salt intake subgroups. Overall, DBP was 77 mmHg (95%CI, 74–81; *I*^2^: 94.7%) and 80 mmHg (95%CI, 76–83; *I*^2^: 93.1%) in low- and high-sodium intake subgroups, respectively; differences in diastolic BP were summarized in Figure 2 with an overall random-effect value of −2.3 mmHg (95%CI: −3.5, −1.2; *p* < 0.001). Heterogeneity was not significant (*I*^2^: 0%, *p* = 0.653). No publication bias was found (Begg’s test: *p* = 0.297; Egger’s test: *p* = 0.331).

A significant difference between low- and high-salt intake was also found in five studies evaluating ambulatory diastolic BP (Figure 2) with an overall random-effect value of −3.0 mmHg (95%CI: −4.3, −1.7; *p* < 0.001). Heterogeneity was not significant among these studies (*I*^2^: 0.3, *p* = 0.405). No publication bias was found (Begg’s test: *p* = 1.0; Egger’s test: *p* = 0.817).

#### 3.3.4. Proteinuria and Albuminuria

**Figure 3:**
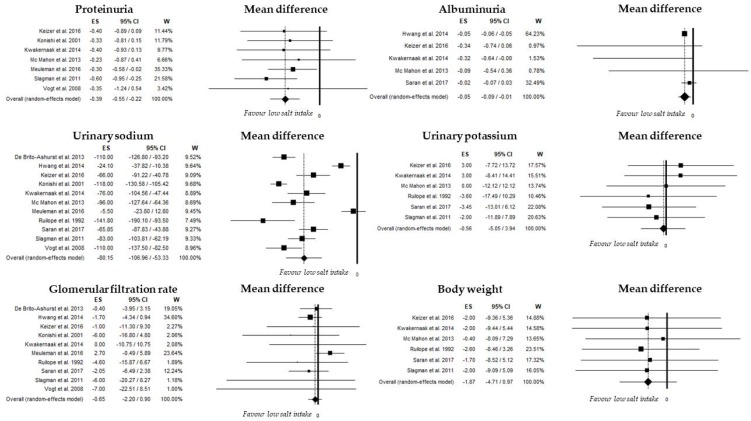
Mean difference of proteinuria, albuminuria, glomerular filtration rate, urinary sodium, urinary potassium and body weight in low- and high-salt intake.

**Figure 4:**
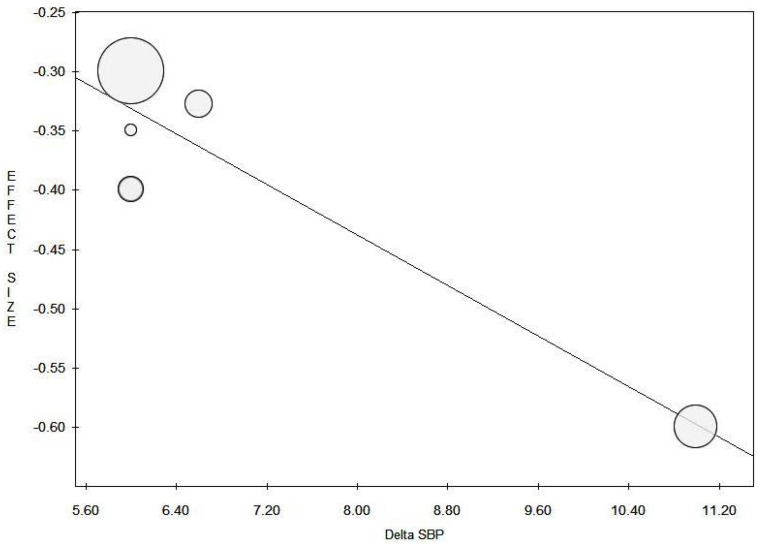
Meta regression of differences in systolic blood pressure and overall unstandardized mean difference of six studies evaluating proteinuria. Y = −0.01 + (−0.05); *p* = 0.005.

Seven studies evaluated difference in proteinuria values comparing low- versus high-salt intake. Overall proteinuria was 0.90 g/day (95%CI, 0.65–1.14; *I*^2^: 81.7%) and 1.34 g/day (95%CI, 1.05–1.63; *I*^2^: 77.7%) in low- and high-sodium intake subgroups, respectively; differences in proteinuria were summarized in Figure 3 with an overall random-effect value of −0.39 g/day (95%CI: −0.55, −0.22; *p* < 0.001). Heterogeneity was not significant (*I*^2^: 0%, *p* = 0.913). No publication bias was found (Begg’s test: *p* = 0.881; Egger’s test: *p* = 0.837). In the evaluation of moderator analyses, we specifically tested the role of SBP differences between low- and high-sodium intake groups on the overall unstandardized mean of proteinuria; we found a significant role (*p* = 0.005), as shown in the meta-regression analysis (Figure 4).

A significant difference between low- and high-salt intake was also found in five studies evaluating differences in albuminuria (Figure 3). Overall albuminuria was 0.23 g/day (95%CI, 0.14–0.31; *I*^2^: 94.7%) and 0.36 g/day (95%CI, 0.23–0.49; *I*^2^: 94.0%) in low- and high-sodium intake subgroups, respectively; differences in albuminuria were summarized in Figure 3 with an overall random-effect value of −0.05 g/day (95%CI: −0.09, −0.01; *p* = 0.013). Heterogeneity was not significant among these studies (*I*^2^: 34.7%; *p* = 0.190). No publication bias was found (Begg’s test: *p* = 1.0; Egger’s test: *p* = 0.477).

#### 3.3.5. Glomerular Filtration Rate

Nine studies evaluated the difference in GFR comparing low- and high-salt intake subgroups; differences in GFR were not significant with an overall random-effect value of −0.7 mL/min (95%CI: −2.2, 0.9; *p* = 0.410) (Figure 3). Heterogeneity was not significant (*I*^2^: 0%, *p* = 0.547). No publication bias was found (Begg’s test: *p* = 0.128; Egger’s test: *p* = 0.158).

#### 3.3.6. Additional Outcomes

We also evaluated the overall unstandardized mean difference of 24-h urinary potassium and body weight comparing low- and high-salt intake subgroups. Differences in UKV were found in six studies and are summarized in Figure 3 with an overall random-effect value of −0.6 mEq/day (95%CI: −5.1, 3.9; *p* = 0.808). Heterogeneity was not significant (*I*^2^: 0%, *p* = 0.922). No publication bias was found (Begg’s test: *p* = 0.851; Egger’s test: *p* = 0.811).

Differences in body weight were found in six studies and are summarized in Figure 3 with an overall random-effect value of −1.9 Kg (95%CI: −4.7, 1.0; *p*: 0.197). Heterogeneity was not significant (*I*^2^: 0%, *p* = 0.999). No publication bias was found (Begg’s test: *p* = 0.091; Egger’s test: *p* = 0.099).

Finally, body fluid composition by bioelectrical impedance spectroscopy (BIS) was evaluated in three studies [26,27,32]. Data could not be meta-analyzed but in these papers, a significant reduction of total body water was found in low salt intake compared with no change in high intake group.

#### 3.3.7. Adverse Effects

We evaluated onset of hypotension and worsening of renal function reported from studies included in the meta-analysis. Six studies did not mention any adverse effect; in the others, one and 17 patients showed severe and mild orthostatic complaints, respectively, during low-sodium diet without drop-out. Four patients showed mild orthostatic complaints during a regular sodium diet. Finally, two subjects dropped out for elevation in serum creatinine during a low-sodium diet but this was not associated with hypotension.

## 4. Discussion

BP control and reduction of proteinuria/albuminuria correction are the cornerstones of CKD management. In this meta-analysis of RCTs in patients with CKD stage 1–4, we demonstrate the efficacy of dietary sodium restriction *per se* in producing a meaningful improvement in clinic and ambulatory systolic and diastolic BP and in proteinuria/albuminuria.

This meta-analysis showed that a moderate dietary sodium restriction from 179 to 104 mEq/day significantly decreased systolic/diastolic BP measured as clinic and ambulatory BP, by 5/2 mmHg and 6/3 mmHg, respectively. The anti-hypertensive effect of salt restriction of our meta-analysis was not dependent on the main demographic and clinical characteristics, such as for example CKD stage and use of anti-RAS inhibitors, since heterogeneity was low and not significant. Indeed, as an exception the occasional patient with renal salt wasting diseases, patients with CKD have a “salt-sensitive” BP phenotype, which may contribute to abnormally high BP and to excess cardiovascular risk [8,33]. Indeed, CKD is characterized by impaired volume homeostasis frequently associated with hypertension [34]. In agreement with this hypothesis, the data on hydration status, though insufficient to allow a pooled analysis, show that after salt restriction, a significant reduction of total body water or extracellular fluid volume ensues. This finding is consistent in all the three studies which measured body fluid composition by bioelectrical electrical impedance [24,26,28]. This secondary hypertension is often resistant to multiple anti-hypertensive drugs likely due to unreduced salt intake [35,36]. Interestingly, similar data were obtained when examining 24-h ambulatory BP that is recognized as the gold standard for BP measurement in CKD [37].

Of note, the beneficial effects of salt restriction may go well beyond the reduction in BP. A low-sodium diet in fact can per se reduce arterial stiffness and left ventricular diastolic dysfunction [38,39], as well as oxidative stress, inflammation and endothelial cell dysfunction [40,41].

More importantly, low-salt decreased 24-h proteinuria and albuminuria by 0.39 g/day and 0.05 g/day, respectively, with respect to higher salt intake. In our meta-analysis, changes in proteinuria were linearly associated with changes in systolic BP, suggesting that the anti-proteinuric effect of sodium restriction may be dependent on BP reduction. On the other hand, in all experimental models of progressive kidney disease characterized by glomerular hypertension and hyperfiltration, proteinuria/albuminuria had been a consistent finding. The direct relationship between changes in systolic BP and changes in proteinuria may be attributed to the impaired renal autoregulation in CKD; accordingly, a greater fall in systolic BP after salt restriction induces a major decrement in the increased glomerular capillary pressure, thus translating in the reduction of proteinuria [42].

Reduction of the main risk factors was generally achieved through a moderate reduction of salt intake without major adverse effects. Occasional severe orthostatic hypotension in fact was detected in one patient while worsening of renal function was reported in two patients without hypotension. Mild orthostatic hypotension was shown in 17 and four patients during low- and high-salt intake, respectively. Therefore, even if adverse effects are uncommon, it is useful to monitor BP also in the orthostatic position in patients adherent to prescription of low-salt diet to eventually downtitrate/withdraw antihypertensive drugs. Finally, some concerns have been recently raised about very low sodium diet (<2 g/day) [43,44]; however, in the studies here examined UNaV levels did not decline below this critical value.

The risk of bias in studies included in the meta-analysis appears to be low and unclear (Table 3). In particular, previous studies had unclear risk while a low-risk was found in more recent papers. The risk of carry-over effect was found in only two papers [23,24], because there was not a washout period between interventions. The greater duration of salt intervention (>4 vs. <4 weeks) significantly attenuates the achievement of salt restriction. Indeed, the long-term sustainability of low-salt diet is the major limitation to the beneficial effects of this dietary intervention. Challenges to optimal implementation include the high sodium content of processed foods, as well as patient-specific barriers (low awareness of the importance of salt intake lowering as the scarce attitudes of individuals in reading the labels of processed food). These problems and the lack of appropriate clinical trials on this issue concur in making dietary salt recommendations inefficacious or poorly utilized in the clinical practice [45]. In this regard, it is valuable to note the information derived from the studies included in this meta-analysis where a low salt intake was generally obtained by educational interventions and replacement of sodium-rich food with low-sodium products. In particular, in the attempt to remove barriers and to detect facilitators to dietary sodium restriction, careful salt intake assessment is essential when treating CKD patients, both in early and advanced stages. According to the results of this meta-analysis, to obtain meaningful improvement in BP and proteinuria control in CKD, it is sufficient to have a moderate reduction of salt assumption with diet, that is equal, on average, to about 110 mEq per day. In this regard, a simple, cheap, colorimetric method by dipstick has been proposed to evaluate the concentration of sodium on urinary samples and, therefore, support self-measurement of the adherence to sodium restricted diets [46].

Our meta-analysis had some limitations. First, RCTs included in this meta-analysis were not so high-quality studies (small sample size, limited patients adherence) and this could affect our results. In particular, regarding proteinuria, recent trials always include patients receiving renin–angiotensin–aldosterone system inhibitors, whereas older studies did not. Second, included trials were mainly performed in East Asia and the Netherland and results could not be generalized. Third, the most important hard renal outcome is the progression of renal function, faithfully represented by changes in eGFR. In this study, there is no significant favor in eGFR change with diet salt restriction. It is basically not applicable because of the short duration of those selected studies and we can draw conclusion about effects on renal progression. Fourth, selected studies covered a wide range of CKD patients (stage 1–4) but when we performed sensitivity analyses, no difference were found in outcome comparing studies with advanced CKD stages with earlier (stages 1–2). Fifth, glomerular filtration rate was estimated and not measured with the gold standard. Therefore, beneficial effects of salt restriction in all patients with CKD may remain inconclusive.

In conclusion, this meta-analysis of RCTs suggests that moderate dietary salt restriction significantly reduces blood pressure and proteinuria, proportionally to BP decline, in patients with early and late CKD with few adverse effects. The main limitation to effective salt restriction is the long-term sustainability. Further studies are therefore needed to test educational tools aimed at optimizing adherence to salt restriction over the long-term. Similarly, long-term RCTs are needed to evaluate the effects on CKD progression and CV outcomes.

## Figures and Tables

**Table 1:** Demographic and clinical characteristics of cohorts included in the systematic review and meta-analysis.

Author/Country (Year)	Nr. Patients Low/High Salt Intake	Study Design	Intervention Duration (Weeks)	Mean Age (Years)	Male Gender (%)	Diabetes (%)	Cardiovascular Disease (%)	Anti-RAS Inhibitors Use (%)	CKD Stage	Interventions	Salt Restriction Modalities	Outcomes
Ruilope/Spain (1992)	14/14	Cross-over RCT	1	NA	NA	0	0	0	1–3	Low salt: 4 g/day High salt: 11 g/day	Salt supplementation in high salt	cSBP, cDBP, aSBP, aDBP, GFR, UNaV, UKV, body weight
Konishi/Japan (2001)	41/41	Cross-over RCT	1	45 ± 15	34.0	0	0	0	1–2	Low salt: 5 g/day High salt: 12 g/day	Replacing sodium-rich products with a low-sodium products	cSBP, cDBP, GFR, proteinuria, UNaV
Vogt/Netherlands (2008)	33/33	Cross-over RCT	18	50 ± 12	75.7	0	0	0	1–3	Low sodium: 50 mmol/day High sodium: 200 mmol/day	Replacing sodium-rich products with a low-sodium product of the same product group	cSBP, cDBP, GFR, proteinuria, UNaV
Slagman/Netherlands (2011)	52/52	Cross-over RCT	6	51	82.7	0	0	100	1–3	Low sodium: 50 mmol/day High sodium: 200 mmol/day	Dietary counseling in low salt	cSBP, cDBP, eGFR, proteinuria, UNaV, UKV, body weight
De Brito-Ashurst/UKV (2011)	25/23	Parallel RCT	24	58	58.3	64.6	NA	NA	3–4	Low salt: educational intervention High salt: regular care	Practical cooking andeducational sessions with dietitian	aSBP, aDBP, eGFR, UNaV, body weight, TBV
McMahon/Australia (2013)	20/20	Cross-over RCT	2	68.5 ± 11	75.0	40	NA	30	3-4	Low salt: 60–80 mmol/day High salt: 180–200 mmol/day	Salt supplementation in high salt	aSBP, aDBP, proteinuria, albuminuria, UNaV, UKV, body weight, TBV
Hwang/Korea (2014)	119/126	Parallel RCT	8	49.5 ± 13.3	49.8	0	0	100	1–3	Low salt: <100 mmol/day High salt: ≥25% reduction from baseline	Intensive and conventional education groups	cSBP, cDBP, GFR, albuminuria, UNaV
Kwakernaak/Netherlands (2014)	45/45	Cross-over RCT	6	65 ± 9	84.0	100	47	100	1–3	Low sodium: 50 mmol/day High sodium: 200 mmol/day	Counselling session with dietitians	SBP, DBP, body weight, GFR, albuminuria, proteinuria, UNaV, UKV
Keizer/Netherlands (2016)	43/44	Cross-over RCT	8	63.2	43.1	0	0	100	1–3	Low sodium: 1.2 g/day High sodium: 4.8 g/day	Replacing sodium-rich products with a low-sodium products	cSBP, cDBP, GFR, proteinuria, albuminuria, UNaV, UKV, body weight
Meuleman/Netherlands (2016)	67/71	Parallel RCT	24	55.1	81.8	25	38	100	1–4	Low salt: self -management intervention High salt: regular care	Nutrition counseling by a dietician and psychologists, point-of-care chip-device	cSBP, cDBP, aSBP, aDBP, GFR, proteinuria, UNaV
Saran/US (2017)	58/58	Cross-over RCT	4	56.5	52.0	38	21	NA	3–4	Low sodium: <2g/day High sodium: 10 g/day	dietary counseling with training in motivational interviewing techniques	aSBP, aDBP, cSBP, cDBP, GFR, albuminuria, UNaV, UKV, body weight, TBV

Abbreviations: NA, not available; cSBP, clinic systolic blood pressure; cDBP, clinic diastolic blood pressure; aSBP, ambulatory systolic blood pressure; aDBP, ambulatory diastolic blood pressure; GFR, glomerular filtration rate; RCT, randomized controlled trial; ClCr, creatinine clearance; MDRD, modification of diet in renal disease formula; RAS, renin angiotensin system; TBV, total body water.

**Table 2:** Renal function parameters, blood pressure and urinary sodium in patients receiving low- or high-sodium diet.

Author		Final GFR(ml/min)	Final Uprot (g/day)	Final Ualb (g/day)	Final Urinary Na (mmol/day)	Final cSBP (mmHg)	Final cDBP (mmHg)	Final aSBP (mmHg)	Final aDBP (mmHg)
Ruilope et al.	Low High	62.7 ± 10.8 * 67.3 ± 18.6 *	NA	NA	72.6 ± 39.1 214.4 ± 83.5	151.6 ± 17.9 154.7 ± 15.8	95.6 ± 10.2 94.9 ± 11.3	146.1 ± 20.2 148.0 ± 21.2	90.3 ± 11.3 90.1 ± 12.1
Konishi et al.	Low High	108 ± 23 * 114 ± 25 *	0.55 ± 0.76 0.88 ± 1.30	NA	48 ± 14 166 ± 37	115 ± 11.2 121.6 ± 13.1	75 ± 8 79 ± 9	NA	NA
Vogt et al.	Low High	82 ± 35 * 89 ± 29 *	2.10 ± 2.10 ^ 2.45 ± 1.55 ^	NA	90 ± 57 200 ± 57	137 ± 17 143 ± 23	83 ± 6 86 ± 11	NA	NA
Slagman et al.	Low High	66 ± 34 * 72 ± 40 *	0.6 ± 0.7 ^ 1.2 ± 1.1 ^	NA	106 ± 50 189 ± 58	123 ± 14 134 ± 22	73 ± 14 80 ± 14	NA	NA
De Brito-Ashurst et al.	Low High	NA	NA	NA	NA	NA	NA	NA	NA
McMahon et al.	Low High	NA	0.64 ± 0.93 0.87 ± 1.12	0.35 ± 0.69 0.44 ± 0.76	82 ± 43 178 ± 58	NA	NA	144.9 ± 13.1 154.6 ± 11.9	79.4 ± 9.4 83.3 ± 9.0
Hwang et al.	Low High	63.4 ± 10.9 # 65.1 ± 10.1 #	NA	0.18 ± 0.01 0.23 ± 0.01	122.2 ± 54.5 146.3 ± 55	121.2 ± 14.2 122.6 ± 14.6	73.6 ± 9.8 74.8 ± 10.1	NA	NA
Kwakernaak et al.	Low High	65 ±27 ° 65± 25 °	0.9 ± 1.0 1.3 ± 1.5	0.39 ± 0.57 0.71 ± 0.93	148 ± 65 224 ± 73	141 ± 16 147 ± 16	79 ± 10 82 ± 10	NA	NA
Keizer et al.	Low High	67 ± 24 # 68 ± 25 #	1.0 ± 1.0 1.4 ± 1.3	0.72 ± 0.8 1.06 ± 1.09	104 ± 59 170 ± 61	123 ± 12 129 ± 14	74 ± 9 77 ± 9	NA	NA
Meuleman et al.	Low High	49.6 ± 9.0 # 46.9 ± 10.1 #	1.1 ± 0.82 1.4 ± 0.84	NA	157.0 ± 52.4 162.5 ± 57.3	133 ± 13.1 139 ± 13.5	81 ± 7.4 83 ± 7.6	128 ± 9.8 130 ± 10.1	75 ± 6.5 77 ± 6.7
Saran et al.	Low High	35.6 # 37.6 #	NA	0.10 0.16	104.8 170.2	127.3 131.4	69.4 70.7	133.5 141.4	71.5 77.3

Abbreviations: NA, not available; GFR, glomerular filtration rate; Uprot, 24-h urinary protein excretion; Ualb, 24-h urinary albumin excretion, cSBP, clinic systolic blood pressure; cDBP, clinic diastolic blood pressure; aSBP, ambulatory systolic blood pressure; aDBP, ambulatory diastolic blood pressure. * glomerular filtration rate measured by creatinine clearance; # glomerular filtration rate estimated by MDRD equation; ° glomerular filtration rate estimated by CKD-EPI equation; ^ proteinuria was measured as protein/creatinine ratio (g/g).

**Table 3:** Risk of bias in studies included in the meta-analysis.

Study	Random Sequence Generation	Allocation Concealment	Blinding of Participants	Blinding of Outcome Assessment	Free of Incomplete Outcome Data	Free of Selective Reporting	Free of Carry Over Effect	Free of bias from Confounders	Free of Others Bias
Ruilope et al.	Unclear	Unclear	Unclear	Unclear	Unclear	Unclear	No	Unclear	Unclear
Konishi et al.	Unclear	Unclear	Unclear	Yes	Unclear	Unclear	No	Unclear	Unclear
Vogt et al.	Yes	Unclear	Unclear	Unclear	Yes	Yes	Yes	No	No
Slagman et al.	Yes	Unclear	Unclear	Unclear	Yes	Yes	Yes	No	Unclear
De Brito-Ashurst et al.	Yes	Unclear	Unclear	Unclear	Yes	Unclear	Yes	Unclear	Yes
McMahon et al.	Yes	Yes	Yes	Yes	Yes	Yes	Yes	Unclear	Yes
Hwang et al.	Yes	Unclear	Unclear	Yes	Yes	Yes	Yes	Unclear	Unclear
Kwakernaak et al.	Yes	Yes	Unclear	Unclear	Yes	Yes	Yes	Yes	Yes
Keizer et al.	Yes	Yes	Unclear	Unclear	Yes	Yes	Yes	Yes	Yes
Meuleman et al.	Yes	Yes	Unclear	Unclear	Unclear	Unclear	Yes	Unclear	Yes
Saran et al.	Yes	Yes	Unclear	Unclear	Yes	Yes	Yes	Yes	Yes

## Appendix A

Item S1. Literature search strategy for PubMed

#1 ((sodium chloride [MeSH Terms]) OR (Diet, sodium restricted [MeSH Terms]))

#2 ((“sodium” [Text Word]) OR (“salt” [Text Word])) AND (low OR high OR alter* OR reduce* OR reduction OR restrict* OR intake* OR diet* OR increas* OR decreas* OR change* OR changing))

#3 (#1 OR #2)

#4 ((“Renal Insufficiency, Chronic”[Mesh Terms]) OR (“Kidney diseases” [Mesh Terms])

#5 ((“chronic kidney disease”[Text Word]) OR (“chronic kidney insufficiency”[Text Word]) OR (“chronic kidney dysfunction”[Text Word]) OR (“chronic renal insufficiency”[Text Word]) OR (“chronic renal dysfunction”[Text Word]) OR (“kidney disease*”[Text Word]) OR (“renal disease*”[Text Word] OR (“nephropath*[Text Word])”OR(“nephrit*”[Text Word]) OR(“glomerulo*” [Text Word]) or (“glomerular disease”[Text Word]))

#6 (#4 OR #5)

#7 (#3 AND #6)

#8 (((Clinical Trial[ptyp] OR Randomized Controlled Trial[ptyp] OR Controlled Clinical Trial[ptyp]) AND “humans”[MeSH Terms])

#9 (#7 AND #8)
